# Health service use and costs associated with aggressiveness or agitation and containment in adult psychiatric care: a systematic review of the evidence

**DOI:** 10.1186/s12888-015-0417-x

**Published:** 2015-03-04

**Authors:** Maria Rubio-Valera, Juan V Luciano, José Miguel Ortiz, Luis Salvador-Carulla, Alfredo Gracia, Antoni Serrano-Blanco

**Affiliations:** Fundació Sant Joan de Déu, Esplugues de Llobregat, Spain; Primary Care Prevention and Health Promotion Research Network (RedIAPP), Barcelona, Spain; School of Pharmacy, Universitat de Barcelona, Barcelona, Spain; Open University of Catalonia (UOC), Barcelona, Spain; Parc Sanitari Sant Joan de Déu, Sant Boi de Llobregat, Spain; Centre for Disability Research and Policy, Faculty of Health Sciences, and Mental Health Policy Unit, Brain and Mind Research Institute, University of Sydney, Sydney, Australia; Scientific Area, Ferrer, Barcelona, Spain; Research & Development Unit, Parc Sanitari Sant Joan de, 22 Déu. C/ Dr. Antoni Pujadas 42, 08830 Sant Boi de Llobregat, Barcelona Spain

**Keywords:** Aggression, Agitation, Containment, Costs, Health services research, Inpatients, Acute psychiatric wards, Review

## Abstract

**Background:**

Agitation and containment are frequent in psychiatric care but little is known about their costs. The aim was to evaluate the use of services and costs related to agitation and containment of adult patients admitted to a psychiatric hospital or emergency service.

**Methods:**

Systematic searches of four electronic databases covering the period January 1998-January 2014 were conducted. Manual searches were also performed. Paper selection and data extraction were performed in duplicate. Cost data were converted to euros in 2014.

**Results:**

Ten studies met inclusion criteria and were included in the analysis (retrospective cohorts, prospective cohorts and cost-of-illness studies). Evaluated in these studies were length of stay, readmission rates and medication. Eight studies assessed the impact of agitation on the length of stay and six showed that it was associated with longer stays. Four studies examined the impact of agitation on readmission and a statistically significant increase in the probability of readmission of agitated patients was observed. Two studies evaluated medication. One study showed that the mean medication dose was higher in agitated patients and the other found higher costs of treatment compared with non-agitated patients in the unadjusted analysis. One study estimated the costs of conflict and containment incurred in acute inpatient psychiatric care in the UK. The estimation for the year 2014 of total annual cost per ward for all conflict was €182,616 and €267,069 for containment based on updated costs from 2005.

**Conclusions:**

Agitation has an effect on healthcare use and costs in terms of longer length of stay, more readmissions and higher drug use. Evidence is scarce and further research is needed to estimate the burden of agitation and containment from the perspective of hospitals and the healthcare system.

**Electronic supplementary material:**

The online version of this article (doi:10.1186/s12888-015-0417-x) contains supplementary material, which is available to authorized users.

## Background

Although there is no universally accepted definition, agitation can be defined as a state of motor restlessness accompanied by mental tension that can be present in medical and psychiatric disorders (e.g., schizophrenia, bipolar disorder, Alzheimer’s disease) [1] or emerge as a stand-alone behavioral problem. It is considered a frequent psychiatric emergency that presents across a wide clinical spectrum: it can evolve from mild psychomotor restlessness and mental tension to overt disruptive, aggressive or violent behavior. Approximately 30% of patients with a first psychotic episode who attend psychiatric services show risk of self-harm or other aggressive behavior [2]. Disturbed or violent behavior in patients in adult inpatient psychiatric units can affect their own safety, that of other patients, and staff. An NHS survey covering 1998/1999 found that there were approximately 65,000 violent incidents against staff across the National Health Service in the UK. The average number of incidents in mental health/learning disability trusts was over three times the average for all trusts [3].

Restraint, seclusion, coercion and containment are frequent health care interventions in psychiatric in-patient settings to manage agitation with disruptive, aggressive or violent behavior [4,5]. In spite of their importance, these clinical actions have received very little attention in the available classifications of health interventions such as the Australian Mental Health Intervention Classification [6] or The Current Procedural Terminology code in the U.S. [7]. “Seclusion” has been defined as “*the placement and retention of an inpatient in a bare room to contain a clinical situation that may result in a state of emergency*” [4]. “Restraint” interventions are designed to “*confine a patient’s bodily movements*” and there are two main subtypes: “physical”, when staff members restrict and hold the patient manually, or “mechanical”; the use of belts, handcuffs, etc., that restrict the patient's movements [4]. “Containment” is a broader term that includes a wide variety of strategies including pharmacological treatment or non-pharmacological interventions or techniques such as increased observation levels, locked wards, de-escalation techniques, use of behavioral agreements or increased staffing levels [5].

Regarding the frequency with which these techniques are used, Stewart and colleagues [8] reviewed 45 studies performed in psychiatric services and observed that there was an average of up to five episodes of restraint per month in wards. The duration of the restraint episodes was about 10 minutes and the factors associated with a greater probability of restraint were: being male, young, and subject to compulsory or involuntary admission. Another review [9] showed great differences between 12 European countries in the type, frequency and duration of containment measures used, with the Netherlands and the UK at either end of the range. For example, a seclusion episode lasts for about 300 hours and mechanical restraint episodes last nearly 1,200 hours on average in the Netherlands; whereas in the UK, seclusion is very infrequent, mechanical restraint is forbidden, and physical restraint lasts considerably less than 30 minutes on average [9]. The EUNOMIA project [10], to date the most extensive study of coercion carried out in European psychiatric inpatient facilities, revealed that the percentage of patients receiving coercive measures (physical restraint, seclusion, and forced medication) in each participating country varied from 21% to 59%. The frequency of use of these measures depended on diagnosis and illness severity but was considerably influenced by the societal attitudes and clinical traditions of each country.

Some systematic reviews have examined the effectiveness of restraint, seclusion, and containment strategies [4,5,11]. Surprisingly, a Cochrane review by Sailas and Fenton [4] pointed out that “*No controlled studies exist that evaluate the value of seclusion or restraint in those with serious mental illness*”. Therefore, no recommendation could be made about the effectiveness, benefit or harmfulness of seclusion or restraint based on scientific evidence. Of the 6 studies reviewed by Muralidharan and Fenton [5], none focused on non-pharmacological methods for containment of aggression or self-harm in people with serious mental illness. Thus, non-pharmacological containment strategies for patients exhibiting disturbed or violent behavior are not supported by evidence from controlled studies. Kynoch et al. [11] reviewed ten studies that evaluated the effectiveness of interventions for preventing and managing aggressive patients in acute hospital settings. The most common interventions for managing aggressive conduct in acute care settings were: staff education programs, chemical restraint and mechanical restraint. Although the three strategies showed some degree of effectiveness, the first was preferred by the authors because it sensitizes staff to the nature of the problem of aggression and develops their knowledge, skills, and attitudes in managing this behavior.

Agitation, and some of the techniques described for its management, have consequences beyond the episode itself. They have been associated with increased length of stay and readmission as well as more use of medication [12]. This increases the burden and management costs associated with hospitalized patients [13]. To the best of our knowledge, no systematic review has been conducted to evaluate the use of services and, consequently, the overall costs of agitation episodes and containment techniques.

The main objective of the present study was to systematically review, for the first time, the evidence on costs and service-use associated with agitation and containment strategies in adult patients with a mental illness admitted to a psychiatric hospital or psychiatric emergency services.

## Methods

PRISMA guidelines for reporting systematic reviews were followed [14]. There was no review protocol.

### Literature search

A systematic review of the literature was performed for studies evaluating the use of services and/or costs of states of agitation and/or aggressiveness and the use of containment measures in adult patients admitted to psychiatric hospitals and/or emergency psychiatric units. Four electronic databases were searched from inception to January 2014: PubMed, CINHAL, ISI Web of Knowledge, and EMBASE. The search strategy used included terms related to 1) episodes of agitation and/or containment, 2) inpatient psychiatric care and 3) use of services and/or costs. The detailed electronic search strategy is displayed in the Additional file 1: Table S1. For the hand search, the reference list of all included citations was reviewed to recover additional articles not identified in the electronic search. Researchers with expertise in the topic of interest were asked to suggest relevant studies.

### Inclusion criteria and study selection

Three researchers screened, in duplicate, the articles identified in the search in two steps: by reviewing the title and abstract of the paper, and by reading the full-text paper. To synthesize the most up-to-date evidence, only papers published in the last 15 years (1998–2014) written in English or Spanish (the languages of the reviewers) were included in the synthesis.

Regarding study design, the inclusion criteria were broad to include as much information as possible. We included any study reporting quantitative data on the use of any service and/or costs related to an episode of agitation and/or containment, independently of the study design. The studies had to include an inpatient adult population with a mental disorder admitted to a psychiatric hospital, the psychiatric service of a general hospital or a psychiatric emergency room. Studies were included if they provided information on a sample of agitated patients. Studies were excluded if they pooled the information on agitated patients with that on non-agitated patients so that information on agitated individuals could not be extracted.

### Quality assessment

Due to the wide range of study designs considered in the review, it was difficult to use a structured quality scale. Furthermore, most of the published checklists and scales are used to evaluate the quality of studies assessing the effectiveness of interventions (randomized or non-randomized controlled trials). Quality was discussed in terms of appropriateness of study design, the quality of the methodological approach and the correct presentation and discussion of results. The quality questions considered and answered for each study are presented in Table 1. Following the quality evaluation, the reviewer classified the studies into five categories ranging from low to high quality.

**Table 1:** **Quality criteria and quality of the included studies**

	**Barlow**	**Carr**	**Compton**	**Flood**	**Jaffe**	**Legris**	**Mellesdal**	**Peiró**	**Putkonen**	**Steinert**
	**2000**	**2008**	**2006**	**2008**	**2009**	**1999**	**2003**	**2004**	**2013**	**1999**
**1. Are the study objectives relevant and well defined?**	Yes	Yes	Yes	Yes	Yes	Yes	Yes	Yes	Yes	Yes
**2. Are the methods of the study appropriate to realize the study objectives?**	Yes	Yes	Retrospective study based on the reviews of clinical charts	The method for estimating costs is based on interviews with key personnel (probability of recall bias)	Retrospective study based on review of clinical charts	Retrospective study based on review of clinical charts	Yes	Retrospective study based on review of clinical charts	Yes	Retrospective study based on review of clinical charts
**3. Were the data collected with sufficient quality (review of patient’s chart, patient interview, missing data…)?**	Yes	Yes	Yes	Yes	Yes	Yes. Some scales had low rates of inter-rater agreement and could not be used in the analysis	Yes	No. High rates of missing data are reported on sociodemographic and clinical variables	Yes	Yes
**4. Was the analysis strategy adequate taking into account the study objectives and methods (statistical methods of analysis well-designed and executed, data adjusted for confounding variables,…)?**	The analysis strategy is adequate but the analyses are not adjusted for confounding variables	Yes	Yes	Yes	Yes	Yes	Yes	The analysis strategy is adequate but the analysis did not take into account missing data and could be biased.	Yes	Yes
**5. Is the presentation of results complete and of good quality (all objectives are addressed, raw and adjusted results are presented, information on variability is presented (i.e., SD, SE or confidence intervals), …)?**	All the objectives are addressed but only raw results are presented. Lacks information on variability	Yes	Yes	Yes	Absence of possible confounding issues in the analyses (e.g., relevant physical-mental comorbidities)	Yes	Yes	28% of data is missing for important variables (age, length of illness)	Yes	Yes
**6. Are the results discussed in the context of previously published studies?**	Yes	Yes	Yes	The results are compared only to those previously obtained in USA	Yes	Yes	Yes	Yes	Yes	Yes
**7. Are the limitations of the study discussed and the results discussed taking into account these limitations?**	The limitations of the study are not adequately discussed	Yes	Yes	Yes	Yes	The limitations of the study are not discussed or taken into account when drawing conclusions	Yes	Yes	Yes	Yes
**8. Are the conclusions of the study supported by the results?**	Yes	Yes	Yes	Yes	Yes	Yes	Yes	Yes	Yes	Yes
**QUALITY**	Low-Moderate	High	High	Moderate-High	Low-Moderate	Moderate	High	Low-moderate	High	High

### Data abstraction

Three reviewers used an abstraction form to extract information on the characteristics, methods and outcomes of the retrieved studies. The abstraction form included information on the study design: setting; characteristics of the study population; total sample size and size of the agitated sample; date of the study; and data related to the use of services and costs. Outcome and variability measures were extracted for the sample of agitated/contained patients and for non-agitated/non-contained patients where possible. Only a qualitative synthesis was carried out due to the scarcity of studies, considerable design differences and heterogeneity of reported outcomes.

To homogenize, cost data originally in euros (€) or British pounds (£) was converted to euros in 2014. First, all costs were updated to the reference year using each country’s annual inflation rates. Subsequently, the currency was converted into euros taking into account purchasing power parity conversion factors. We used the annual inflation rates and purchasing power parity conversion factors employed by the Organization for Economic Co-operation and Development (OECD) [15].

## Results

### Literature search and study selection

**Figure 1:**
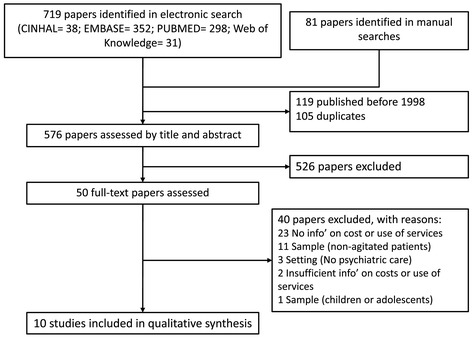
**Flow diagram.**

Figure 1 shows the flow diagram of studies identified in the search and screened for inclusion. The search of electronic databases identified 719 studies and the hand search a further 81 studies. One-hundred and five studies were duplicates and 119 were published before 1998. Of the remaining 576 studies, 526 were excluded after a review of the title and abstract and 40 following a review of the full-text paper (23 did not include information about costs or use of services; 11 did not consider a sample of agitated patients; 3 used a setting other than a psychiatric hospital or unit; and 1 considered an infant or adolescent population). Two papers [16,17] included some information about use of services but the focus of the studies was to define the types and frequency of agitation management and predictors of agitation/restraint, while information on use of services and costs was limited. One of the studies only showed the proportion of patients receiving distinct types of treatments and the proportion of patients requiring more than one drug [17]. The second study provided information on the frequency of mechanical and pharmacological restraints used separately or in combination and the duration of mechanical restraint episodes. Finally, ten papers were included in the synthesis [16].

### Study quality

Table 1 shows the quality of the 10 studies included in the review [11,18-26]. Five studies were considered of high quality, 1 of moderate-high quality, 1 of moderate quality and 3 of moderate-low quality. The main reasons for receiving a “moderate-low” evaluation were as follows: missing data; missing information on relevant results; unsuitable analysis strategy or lack of adjustment for confounding variables in the analysis; and lack of information on variability in the presentation of results.

### Study characteristics

Table 2 shows the characteristics of the studies included. Most had been conducted in Australia (2) and the USA (2) while others had been carried out in Canada, Finland, Germany, Norway, Spain and the UK. The majority of the studies were retrospective cohorts based on the review of clinical charts (5) or prospective cohorts (3). One study was an epidemiological, cross-sectional study based on questionnaires completed by nurses and one was a cluster-randomized controlled trial. The follow-up periods ranged from 6 months to 4 years.

**Table 2:** **Characteristics of the included studies**

**Paper**	**Country and period of study (year)**	**Design**	**Setting**	**Diagnosis (%)**	**Mean age**	**% of men (N)**	**Total sample size**	**Agitated sample size**
Barlow K et al. 2000 [18]	Australia, 18 months (1996–1997)	Prospective observational study	3 acute psychiatric units and 1 subacute unit	Schizophrenia (21.3)	37 (range: 13–97)	52.2 (663)	1,096	174
				Psychotic disorder (14.0)				
				Bipolar affective (7.2)				
				Adjustment disorder (18.5)				
				Depression (18.5)				
				Personality disorder (3.0)				
				Anxiety disorder (5.0)				
				Other (12.4)				
Carr VJ et al. 2008 [19]	Australia, 12 months (n.a.)	Prospective observational study	11 mental health units (8 general psychiatric units, 2 high-dependency units and 1 specialized unit)	Drug and alcohol disorder (44.6)	37.1 (SD 14.4)	55.0 (weighted percentage) (2,210)	3,242	849 (Aggressive incidents: 363; Less serious aggressive incidents: 486)
				Schizophrenia or related disorder (38.0)				
				Depression (25.3)				
				Personality disorder (18.9)				
				Adjustment disorder (14.2)				
				Bipolar disorder (14.0)				
Compton MT et al. 2006 [20]	USA, 7 months (2003–2004)	Retrospective database cohort study	2 inpatient psychiatric units (a crisis stabilization unit (CSU) and a longer-stay milieu unit (LSMU))	Schizophrenia or other psychotic disorders (65.0)	LSMU: 37.4 (SD 12.6) CSU: 40.8 (SD 11.8)	LSMU: 40.7 (60), CSU: 48.9 (43)	LSMU: 146; CSU: 88	n.a.
				Unipolar depression (15.8)				
				Bipolar disorder (12.4)				
				Anxiety disorders (0.9)				
				Substance-related disorders (4.3)				
				Other (1.3)				
Flood C et al. 2008 [13]	United Kingdom, 1 year (2005)	Epidemiological, cross-sectional study	136 adult acute inpatient psychiatric wards	n.a.	n.a.		n.a.	
Jaffe A et al. 2009 [21]	USA, 6 months (2005)	Retrospective database cohort study	17 state-run adult civil facilities	Schizophrenia (41.4)	41.5 (SD 13.6)	63 (257)	1,673	415
				Schizoaffective (31.9)				
				Bipolar (10.4)				
				Depression (5.7)				
				Other (10.6)				
Legris J et al. 1999[22]	Canada, n.a. (n.a.)	Retrospective database cohort study	Urban general hospital with two adult psychiatric wards and a special care unit	Schizophrenia (54)	41 (SD 16)	41 (35)	85	41
				Bipolar or schizoaffective (23)				
				Psychotic depression (12)				
				Other (11)				
Mellesdal L 2003 [23]	Norway, 3 years (1997–2000)	Prospective observational study	Psychiatric acute ward	Affective disorders (34.7)	41.1 (SD 15.5)	51 (476)	934	98
				Schizophrenic disorders (19.2)				
				Alcohol/substance abuse (10.4)				
				Personality disorders (9.4)				
				Neurotic/stress-related somatoform disorders (7.1)				
				Other (19.2)				
Peiró S et al. 2004 [24]	Spain, 6 months (1999–2001)	Retrospective database cohort study	Acute inpatient units from 8 general hospitals and psychiatric hospitals	Schizophrenic disorders (64.0)	39.2 (SD 14.5)	44.8 (56)	200	175
				Affective psychosis (22.0)				
				Paranoid states (4.5)				
				Other non-organic psychosis (8.5)				
				Transient organic psychosis (1.0)				
Putkonen A et al. 2013 [26]	Finland, 6 months (2009)	Cluster-randomized controlled trial	Four high-security wards for men with psychotic illness.	Psychotic illness with a history of severe violence (100)	Control group: 40.0 (SD 10.6) Intervention group: 38.4 (SD 10.6)	100	Control group: 930–1,003 patient-days per month (38 beds) Intervention group: 1,306-1,400 patient-days per month (50 beds)	n.a.
Steinert T et al. 1999 [25]	Germany, 4 years (1990–1993)	Retrospective database cohort study	Psychiatric hospital	Paranoid type schizophrenia (78.9)	32.4 (SD 12.7)	55.8 (77)	138	96
				Schizoaffective disorder (15.9)				
				Accompanying substance abuse (29.7)				
				Accompanying somatic disease (21.0)				

The proportion of men and women was relatively balanced in all studies but one [26]. Mean age ranged from 32.4 to 41.5 years and total sample size and size of the agitated sample ranged from 85 to 3,232 and from 41 to 849, respectively. One study presented information on overall costs of agitation [11] while nine studies presented information on the use and/or costs of specific services. Six studies compared samples of aggressive and/or agitated and non-aggressive and non-agitated patients [18,19,21,23-25]. These studies presented information on use of service in terms of lengths of stay (6), readmission rates (4) and cost of medication (1). Two studies assessed the impact of use of seclusion or restraint on length of stay (2) and use of medication (1) [20,22]. One study reported on the number of staff sick days before and after an intervention strategy to reduce the use of coercive measures [26].

### Impact of aggressiveness and/or agitation

Steinert and cols. [25] conducted a retrospective database cohort study in one psychiatric hospital in Germany. The sample consisted of 96 patients showing aggressive or agitated behavior (threats of violence or violence against persons or objects) and 42 non-agitated/aggressive patients. The study evaluated the associations between aggressive behavior and length of stay and readmission. Aggressive behavior showed no association with the length of stay. However, the number of hospitalizations was significantly predicted by aggressive behavior against others (*β* = 0.16, p = 0.03) and against self (*β* =0.19, *p* = 0.01).

The prospective observational study by Barlow and colleagues [18] compared a sample of aggressive patients (“defined as [those engaging in] an act of verbal or physical aggression directed to self or others, irrespective of outcome”) (n = 174) with non-aggressive patients (n = 922) from three acute psychiatric units and one subacute unit in Australia. Length of stay and number of readmissions were evaluated. Mean length of stay was 24.88 (SD not reported) days in the aggressive group and 12.06 (SD not reported) days in the non-aggressive group. The difference in mean days of stay between groups was statistically significant (F = 68.34; p < 0.001). The mean number of readmissions was 3.56 (SD not reported) and 1.75 (SD not reported) in the aggressive versus the non-aggressive sample, respectively. The study reported a statistically significant difference in the mean number of readmissions over the 18-month period for the aggressive versus non-aggressive patients (F = 125.22, p < 0.001).

Mellesdal and cols. [23] conducted a prospective observational study in a psychiatric acute ward in Norway. The study compared patients with (n = 98) and without (n = 836) aggressive incidents (“behavior intended to cause bodily harm or physical injury [to] other persons and as verbal and physical threats of inflicting bodily harm upon others”) and compared mean length of stay and mean number of readmissions between groups. The mean length of stay for aggressive patients was significantly higher than for non-aggressive patients (32.6 vs 9.7, p < 0.01). The mean number of readmissions was also significantly higher than for non-aggressive patients (2.5 vs 1.5, p < 0.01) with a significantly higher proportion of readmissions of aggressive patients (*χ*^2^ = 53.2, p < 0.001).

Peiró and cols. [24] conducted a retrospective cohort study with databases from acute inpatient units from 8 general hospitals and psychiatric hospitals. The study evaluated the relationship between the presence of aggressiveness and/or agitation (“defined as an annotation in the medical record of this symptom”) (n = 175) and not presenting aggressiveness and/or agitation (n = 25), and the length of stay and the cost of medication. The mean length of stay in the group of patients presenting aggressiveness and/or agitation was 21.87 days (95% CI 18.88, 24.87) while it was 21.08 days (95% CI 14.52, 27.64) in the patients not presenting aggressiveness and/or agitation. No statistically significant association between aggressiveness and/or agitation and length of stay was observed. Mean antipsychotic drug cost was 96.76€ (95% CI 72.5€, 121.0€) for aggressive and/or agitated patients and 23.47€ (95% CI 5.0€, 42.0€) for non-aggressive and non-agitated individuals. The difference in the extra mean cost of treatment was statistically significant only in the unadjusted analysis (p < 0.05) while the updated mean extra cost of the treatment of agitated patients was 50.97€ (95% CI −28.1€, 130.0€; p = 0.171) in the adjusted analysis.

The study by Carr and cols. [19] was a prospective observational study including a sample of 3,242 patients from 11 mental health units in Australia. The study evaluated two groups of patients: patients involved in serious aggressive incidents (“i.e., involving physical contact or a definite intention to inflict harm”) (n = 363) and patients involved in less serious aggressive incidents (“i.e., verbal threats or demands without a plan to inflict harm”) (n = 486). The study evaluated the association between aggressive status and length of stay, and number of readmissions. Mean length of stay was 27.34 (SD 28.61) days for patients involved in aggressive incidents and 14.38 (SD 17.57) days for those not involved. In patients involved in less serious aggression and those not involved in any aggressive incidents, mean length of stay was 23.30 (SD 23.30) and 14.54 (SD 18.90) days, respectively. Reported serious aggressive incidents were strongly associated with length of stay both in the unadjusted and adjusted analyses, with a mean of 12.96 and 11.68 extra days (p < 0.001), respectively. This was also the case for less serious aggression, with a mean of 8.77 and 8.28 extra days in the unadjusted and adjusted analyses, respectively (p < 0.001). The proportion of readmitted patients within 28 days after discharge was higher in the group of patients involved in serious aggressive incidents (21.6%) than in the group of those not involved (14.8%), both in the unadjusted (OR 1.59 p < 0.001) and adjusted analyses (OR 1.74 p < 0.001). In the group of patients involved in less serious aggressive incidents, readmission occurred in 19.3% compared with 14.9% in the group of patients not involved. However, this difference was not statistically significant in either the adjusted or unadjusted analysis.

Jaffe and cols. [21] reviewed, in a retrospective cohort study, the database of 17 state-run adult civil facilities. They assessed length of stay (time to discharge) in a sample of 415 agitated patients (“patients receiving an intramuscular ”stat“ medication order for the presumed treatment of agitation”) and 1,258 non-agitated patients (“patients who did not have an order for an ’agitation stat”). The adjusted odds ratio for discharge at 6 months was 0.63 (95% CI 0.46, 0.86, p = 0.004) for agitated compared with non-agitated patients. Median time to discharge in the agitated and non-agitated sample was 164 days (95% CI 129, 199) and 110 days (95% CI 100, 120), respectively, with a relative risk difference for discharge within the first 30 days of 0.55 (95% CI 0.33, 0.90).

Putkonen and cols. [26] conducted a cluster-randomized trial in 4 wards in Finland to evaluate the effectiveness of preventing coercive measures without violence in high-security wards for males with schizophrenia and a history of severe violence. The program was implemented in 2009. One of the outcome measures was length of staff sick-leave time after a patient-to-staff injury. The mean duration of sick leave was 8.8 days per injury in 2007, 1.6 days per injury in 2008 and 1.8 days per injury in 2009.

### Impact of seclusion and/or restrain

Legris and cols. [22] conducted a retrospective cohort study using the databases of one urban general hospital with two adult psychiatric wards and a special care unit comparing cohorts of secluded (41) and non-secluded (44) patients. Mean length of hospital stay was 41 (SD 31.0) days for secluded patients and 29 (SD 24.8) days for non-secluded patients. This difference did not reach statistical significance (p = 0.053). There was a higher mean daily drug dose (in chlorpromazine equivalents) in the secluded sample (748.5 mg) compared with the non-secluded sample (464.8 mg) (p = 0.036).

The study by Compton and cols. [20] was a retrospective database cohort study using data from two inpatient psychiatric units in the USA: a crisis stabilization unit (CSU) and a longer-stay milieu unit (LSMU). The study included 88 patients from the CSU and 146 patients from the LSMU. The use of seclusion or restraint was not associated with a longer length of stay among patients admitted to the CSU (Beta = 1.22, SE = 0.71, p = 0.09) but it was among patients admitted to the LSMU (Beta (log10) = 0.16, SE = 0.01, p < 0.01), with a mean of 1.45 (95% CI 1.21, 1.73) extra days of stay.

### Costs of conflictive behaviors and containment

One study conducted in the UK evaluated the national costs of conflictive behaviors and containment in psychiatric acute units in 2005 using an epidemiological, cross-sectional study [11]. Table 3 shows the aggregated and updated costs per ward for the conflictive behavior and containment events considered in the study. Conflictive behavior included verbal abuse; aggression towards objects or persons; self-harm; refusing to follow indications; use of alcohol or drugs; absconding; and refusing medicines. The total cost per ward estimated for the year 2014 of all conflictive behavior was €182,616 while the total national cost was 91 million euros. The containment events included use of medication; referral to intensive care units; observation; show of force, restraint and seclusion; and time out. The total containment costs per ward were €182,616 while national containment costs reached 133 million euros. Verbal abuse, aggression (to objects, others and self) accounted for an elevated proportion of the costs of conflictive behavior (€47,529 per ward). The most expensive containment event was intermittent observation and special observation at €204,072 per ward.

**Table 3:** **Aggregated conflict behaviors and containment event updated annual costs [and original costs (11)]**

**Conflict behaviors costs**	**Cost per ward**	**Cost nationally**
Verbal abuse, aggression towards objects and physical assault	47,529€ [£37,785]	23,764,980€ [£18,892,816]
Self harm	10,284€ [£8,176]	5,142,434€ [£4,088,161]
Smoking in non-smoking areas or refusing to eat, drink, wash, get up, go to bed or see workers	55,319€ [£43,978]	27,659,246€ [£21,988,701]
Alcohol or drug use	11,565€ [£9,194]	5,782,210€ [£4,596,773]
Attempts to abscond or absconding	31,807€ [£25,286]	15,903,323€ [£12,642,912]
Refused regular or *Pro Re Nata* medicines or demand of *Pro Re Nata* medicines	26,112€ [£20,759]	13,055,998€ [£10,379,330]
Cost of all conflict behavior	182,616€ [£145,177]	91,308,194€ [£72,588,694]
**Containment events costs**	**Cost per ward**	**Cost nationally**
Given *Pro Re Nata* or intramuscular medicines	28,916€ [£22,988]	14,457,777€ [£11,493,724]
Sent to Intensive Care Unit or Intermediate Care Area	2,549€ [£2,026]	1,274,497€ [£1,013,207]
Intermittent and constant special observation	204,072€ [£162,234]	102,035,487€ [£81,116,737]
Show of force, manual restraint and seclusion	28,518€ [£22,671]	14,259,142€ [£11,335,812]
Time out	3,015€ [£2,397]	1,507,595€ [£1,198,516]
Cost of all containment events	267,069€ [£212,316]	133,534,500€ [£106,157,997]

## Discussion

Six of the 8 studies included in our review that evaluated length of stay found a positive association between length of stay and agitation states or containment [18-23], while all of the studies that evaluated the probability of readmission reported a statistically significant association with aggressiveness or agitation [18,19,23,25]. Two studies examined the use of drugs in agitated and secluded patients by comparing mean daily medication dose and mean cost of treatment [22,24]. These studies found that medication doses and costs were associated with seclusion and agitation, respectively.

The results of our systematic review can be summarized as follows: first, the evidence on use of services and costs of agitation and containment in inpatient psychiatric care was scant. Only ten studies from the last 15 years provide information on use of services and costs, and very few services were evaluated. Second, the nine study designs differed widely, complicating between-study comparisons. Methodological quality was high or moderately-high in 5 of the 9 studies. Third, according to the retrieved studies, aggressiveness, agitation and containment measures are associated with increased services and costs (in terms of length of stay, higher readmission rates and increased medication consumption). Evidence on readmission and use of medicines is inconclusive. However, the studies that did not identify a statistically significant difference in length of stay between agitated/aggressive and non-agitated/aggressive patients were those using a higher threshold for behavior defined as agitated or aggressive. One study reported that the mean duration of staff sick leave after an incident of patient aggression ranged from 1.6 to 8.8 days per injury but the study did not compare samples of agitated and non-agitated patients. Finally, only one study focused on the annual economic consequences of conflictive behaviors and containment events; found to be over €91 and €133 million, respectively, in the UK.

As shown in previous reviews [27], several factors, particularly context and patient characteristics, influence seclusion. A similar conclusion can be inferred regarding restraint. In this area of research, the famous “chicken and egg dilemma” remains unresolved. There appear to be various possibilities that are not mutually exclusive: (1) higher agitation might be a “causal” factor of an increased length of stay because it may itself be a sign of illness severity; (2) a longer stay in an aversive or restrictive environment may cause increased levels of agitation, aggression, or absconding; or (3) the significant relationship between the agitation and length-of-stay variables might be mediated by unknown variables linked to the context where this relationship is observed. Future studies should focus on the causal association between these variables and go beyond correlational analysis.

Most of the retrieved studies focused on direct healthcare costs from the perspective of the hospital and did not take into account other costs such as those related to patient and staff productivity losses. Furthermore, most of the studies only evaluated whether agitated or aggressive behaviors were associated with increased use of some services and/or costs but did not quantify the costs. This might suggest that the optimal design for measuring the costs of agitation is a cost-of-illness study [28] that takes into account its prevalence. Ideally, such a study should consider all relevant services and costs, including indirect costs such as those related to premature death. Intangible costs related to patient suffering are also relevant although measuring them is troublesome. Differences exist between countries in use and duration of containment so multicenter international studies should be encouraged. This would facilitate better understanding of the issue and allow informed decision-making regarding the efforts required to prevent and manage these episodes. With the available evidence, the optimal strategy appears to be a reduction of special observation events.

To allow comparisons between studies, it is essential to reach consensus on the definition and operational criteria of what is considered to be an agitation episode, as well as a standard definition and taxonomy for the related care interventions. The study by Flood et al. [13] considers conflictive behaviors and includes costs resulting from a patient refusing to eat, drink or wash, whether or not this ends in verbal abuse or aggression. A number of studies considered aggressiveness or episodes of aggression while others focused on more severe cases such as secluded patients. This adds heterogeneity to the study results. Some instruments for quantifying the nature and frequency of aggressive behaviors exist such as the Report Form for Aggressive Episodes (REFA) [29] or the Staff Observation Aggression Scale [30]. It is vital to bear the nature of the case in mind when conducting a cost-of-illness study [28]. However, a cost-of-illness study will only provide information on the extent of the problem in terms of impact on healthcare resources and labor productivity [28]. Economic evaluations of interventions designed to improve inpatient care and reduce costs resulting from aggressiveness and agitation must be conducted to support their implementation.

Rates of containment and restraint could be relevant indicators of both the quality of individual services and the quality and performance of the mental health system as a whole. However, to be effectively used for evidence-informed policy, these rates need to be contextualized in relation to the number of residential services available for acute care, the placement capacity in the system, outpatient acute services and other data on service availability. Even when national data are available (e.g., Finland and Norway) [8], these may not indicate system quality unless full information is available on the number of beds for acute care in the system. Taking the Roemer effect into account (hospitalization depends on bed availability more than on severity of illness) [31], higher rates of restraint and constraint could be due to greater availability of acute beds in the system and not to lower quality of acute care. Unfortunately, none of the published studies on constraint and containment provided comparable information on service availability. Therefore, health-action categorization systems, such as the International Classification of Health Interventions (ICHI), should be complemented with the use of standard classifications of acute care services such as that provided in the context of long-term care by the DESDE-LTC system [32]. Future studies should provide more detailed information, especially on service availability at the local or national level [33].

The effectiveness of seclusion and restraint techniques is not supported by empirical evidence [3,4]. Indeed, the reduction or elimination of these procedures should be associated with savings and cost-benefits by reducing staff and patient injuries, workers’ compensation costs and claims, liability savings, lost staff time and associated expenses, staff turnover, and staff absenteeism. Shorter lengths of stay and a decrease in rehospitalization rates are also very important benefits [34]. Moreover, seclusion and restraint can have severe psychological and physical consequences for all individuals involved [35], a high level of patient-perceived coercion can decrease treatment satisfaction [36] and being restrained can even decrease the likelihood of attending further follow-up and treatment sessions [37]. A body of work has been developed with the aim of replacing these techniques with more adaptive strategies [38,39]. One recent approach is the de-escalation intervention described in the BETA project [40]. Another interesting intervention, when de-escalation techniques have failed, is the TREC-SAVE study [41] where researchers suggest the least restrictive option for aggressive/agitated psychotic patients when admitted to emergency rooms. Nonetheless, although these new alternatives seem effective in reducing seclusion and restraint, some important issues remain unexplored: can these new interventions increase quality of life and wellbeing? Are these cost-saving interventions? Can they decrease length of stay and use of psychiatric services?

Our results should be interpreted with the following limitations in mind. We only considered studies conducted in an adult population receiving inpatient care and only included papers published in the last fifteen years. This increases the homogeneity and current relevance of the results but limits extrapolation to other populations and contexts. Heterogeneity of the definitions of agitation and aggressive behaviors used in all the studies included could increase variability of results and hamper comparisons. We did not search the grey literature and only included papers written in English or Spanish. However, we searched four distinct electronic databases and conducted an intensive manual search. In addition, we accepted any study design to identify all the relevant literature on the topic. The scale used to evaluate quality was not a structured-quality scale but enabled us to score and compare research using various study designs. Study selection, data extraction and quality assessment were done in duplicate to minimize bias.

## Conclusions

Agitation and/or aggressiveness are relevant components of hospital costs but the evidence on expenditure is scarce. The absence of consensus on the definition of agitated and/or aggressive behavior, together with the heterogeneous evaluation methods, limit our capacity to draw conclusions. Furthermore, the studies retrieved focused on use of services and/or costs from the perspective of the hospital or psychiatric service and only one study evaluated the impact of agitation and/or aggressive behavior on indirect costs. The present systematic review has identified some gaps in this area of research that need to be addressed. Commonly accepted terminology and taxonomy is required and more studies are needed, particularly with respect to causal relationships and the issue of how to standardize study designs to allow international comparison of results. It is to be expected that different types of restraint incur different costs, which should also be evaluated. Interventions aiding the management of aggressive incidents should be coded and classified. To serve as reliable performance and quality indicators, contextual information on service availability should also be provided.

## Additional file

Additional file 1: Table S1. Detailed search strategy in the four electronic databases.
