# Supplementary material for: Health service use and costs associated with aggressiveness or agitation and containment in adult psychiatric care: a systematic review of the evidence
Source: BMC Psychiatry. 2015 Mar 4;15:35. doi: 10.1186/s12888-015-0417-x (PMC4356166; doi:10.1186/s12888-015-0417-x)
Supplement: Additional file 1: Table S1. — Detailed search strategy in the four electronic databases. [file 12888_2015_417_MOESM1_ESM.doc]

| **PubMed** |
| --- |
| ("Violence"[Mesh] OR "Crisis Intervention"[Mesh] OR "Psychomotor Agitation"[Mesh] OR "Agression*" OR "Agressiveness" OR "Agitation" OR "Crisis Interventions" OR "Crisis Intervention" OR "Psychomotor Hyperactivity" OR "Psychomotor Agitation" OR "Psychomotor Excitement" OR "Psychomotor Restlessness" OR "Akathisia" OR " Workplace Violence" OR "Assaultive Behavior" OR "Assaultive Behaviour" OR "Aggression*" OR "Restraint, Physical"[Mesh] OR "Immobilization"[Mesh] OR restrain* OR seclusion OR coercion OR coercive*) AND ("Emergency Services, Psychiatric"[Mesh] OR "Hospitalization"[Mesh] OR "Psychiatric Department, Hospital"[Mesh] OR "Hospitals, Psychiatric"[Mesh] OR "Psychiatric Unit" OR "Psychiatric Units" OR "Psychiatric Emergency Services" OR "Psychiatric Emergency Service" OR "Hospitalization*" OR "Psychiatric Departments" OR "Psychiatric Department" OR "Psychiatric Hospitals" OR "Psychiatric Hospital" OR "Mental Institutions" OR "Mental Institution" OR "Mental Hospital" OR "Mental Hospitals") AND ("Health Services Research"[Mesh] OR"Health Care Costs"[Mesh] OR"Cost of Illness"[Mesh] OR "Health Services Evaluation" OR "Health Services Evaluations" OR "Healthcare Cost" OR "Healthcare Costs" OR "Health Care Cost" OR "Health Care Costs" OR "Health Cost" OR "Health Costs" OR "Medical Care Costs" OR "Medical Care Cost" OR "Service Cost" OR "Service Costs" OR "Use of services" OR "Care Use" OR "Use of Health Services" OR "Healthcare Utilization" OR " Health Care Utilization" OR "Health-Service Utilization" OR "Healthcare Use" OR " Health Care Use" OR "Health-Service Use") |
| **CINHAL** |
| ("Violence" OR "Crisis Intervention" OR "Psychomotor Agitation" OR "Aggressiveness" OR "Agitation" OR "Crisis Interventions" OR "Psychomotor Hyperactivity" OR "Psychomotor Agitation" OR "Psychomotor Excitement" OR "Psychomotor Restlessness" OR "Akathisia" OR " Workplace Violence" OR "Assaultive Behavior" OR "Assaultive Behaviour" OR "Aggression*" OR "Restraint" OR "Immobilization" OR restrain* OR seclusion ) AND ("Health Services Research" OR "Health Care Costs" OR "Cost of Illness" OR "Health Services Evaluation" OR "Health Services Evaluations" OR "Healthcare Cost" OR "Healthcare Costs" OR "Health Care Cost" OR "Health Cost" OR "Health Costs" OR "Medical Care Costs" OR "Medical Care Cost" OR "Service Cost" OR "Service Costs" OR "Use of services" OR "Care Use" OR "Use of Health Services" OR "Healthcare Utilization" OR " Health Care Utilization" OR "Health-Service Utilization" OR "Healthcare Use" OR " Health Care Use" OR "Health-Service Use") AND ("Emergency Services, Psychiatric" OR "Hospitalization" OR "Psychiatric Department, Hospital" OR "Hospitals, Psychiatric" OR "Psychiatric Unit" OR "Psychiatric Units" OR "Psychiatric Emergency Services" OR "Psychiatric Emergency Service" OR "Hospitalization*" OR "Psychiatric Departments" OR "Psychiatric Department" OR "Psychiatric Hospitals" OR "Psychiatric Hospital" OR "Mental Institutions" OR "Mental Institution" OR "Mental Hospital" OR "Mental Hospitals") |
| **ISI Web of Knowledge** |
| TOPIC: ("Violence" OR "Crisis Intervention" OR "Psychomotor Agitation" OR "Aggressiveness" OR "Agitation" OR "Crisis Interventions" OR "Psychomotor Hyperactivity" OR "Psychomotor Agitation" OR "Psychomotor Excitement" OR "Psychomotor Restlessness" OR "Akathisia" OR " Workplace Violence" OR "Assaultive Behavior" OR "Assaultive Behaviour" OR "Aggression*" OR "Restraint" OR "Immobilization" OR restrain* OR seclusion) AND TOPIC: ("Health Services Research" OR "Health Care Costs" OR "Cost of Illness" OR "Health Services Evaluation" OR "Health Services Evaluations" OR "Healthcare Cost" OR "Healthcare Costs" OR "Health Care Cost" OR "Health Cost" OR "Health Costs" OR "Medical Care Costs" OR "Medical Care Cost" OR "Service Cost" OR "Service Costs" OR "Use of services" OR "Care Use" OR "Use of Health Services" OR "Healthcare Utilization" OR " Health Care Utilization" OR "Health-Service Utilization" OR "Healthcare Use" OR " Health Care Use" OR "Health-Service Use") AND TOPIC: ("Emergency Services, Psychiatric" OR "Hospitalization" OR "Psychiatric Department, Hospital" OR "Hospitals, Psychiatric" OR "Psychiatric Unit" OR "Psychiatric Units" OR "Psychiatric Emergency Services" OR "Psychiatric Emergency Service" OR "Hospitalization*" OR "Psychiatric Departments" OR "Psychiatric Department" OR "Psychiatric Hospitals" OR "Psychiatric Hospital" OR "Mental Institutions" OR "Mental Institution" OR "Mental Hospital" OR "Mental Hospitals")  Timespan=All years. Indexes=SCI-EXPANDED, SSCI, A&HCI, CPCI-S, CPCI-SSH, CCR-EXPANDED, IC. |
| EMBASE |
| (("Violence" OR "Crisis Intervention" OR "Psychomotor Agitation" OR "Aggressiveness" OR "Agitation" OR "Crisis Interventions" OR "Psychomotor Hyperactivity" OR "Psychomotor Agitation" OR "Psychomotor Excitement" OR "Psychomotor Restlessness" OR "Akathisia" OR " Workplace Violence" OR "Assaultive Behavior" OR "Assaultive Behaviour" OR "Aggression*" OR "Restraint" OR "Immobilization" OR restrain* OR seclusion OR coercion* OR coercive*) and ("Health Services Research" OR "Health Care Costs" OR "Cost of Illness" OR "Health Services Evaluation" OR "Health Services Evaluations" OR "Healthcare Cost" OR "Healthcare Costs" OR "Health Care Cost" OR "Health Cost" OR "Health Costs" OR "Medical Care Costs" OR "Medical Care Cost" OR "Service Cost" OR "Service Costs" OR "Use of services" OR "Care Use" OR "Use of Health Services" OR "Healthcare Utilization" OR " Health Care Utilization" OR "Health-Service Utilization" OR "Healthcare Use" OR " Health Care Use" OR "Health-Service Use") and ("Emergency Services, Psychiatric" OR "Hospitalization" OR "Psychiatric Department, Hospital" OR "Hospitals, Psychiatric" OR "Psychiatric Unit" OR "Psychiatric Units" OR "Psychiatric Emergency Services" OR "Psychiatric Emergency Service" OR "Hospitalization*" OR "Psychiatric Departments" OR "Psychiatric Department" OR "Psychiatric Hospitals" OR "Psychiatric Hospital" OR "Mental Institutions" OR "Mental Institution" OR "Mental Hospital" OR "Mental Hospitals")).mp. [mp=title, abstract, subject headings, heading word, drug trade name, original title, device manufacturer, drug manufacturer, device trade name, keyword]  Timespan= 1998-2014. |
